# Follow the protocol and kickstart the heart—Intensive care nurses’ reflections on being part of rescue situations in interdisciplinary teams

**DOI:** 10.1002/nop2.1050

**Published:** 2021-08-25

**Authors:** Annakarin Olsson, Fredric Sjöberg, Martin Salzmann‐Erikson

**Affiliations:** ^1^ Department of Caring Sciences Faculty of Health and Occupational Studies University of Gävle Gävle Sweden; ^2^ Unit of Anaesthesiology and Intensive Care Södersjukhuset Karolinska University Hospital Stockholm Sweden

**Keywords:** cardiopulmonary resuscitation, critical care, emergency team, experiences, interdisciplinary team, qualitative research, registered nurses

## Abstract

**Aim:**

To describe intensive care nurses' reflections on being part of interdisciplinary emergency teams involved in in‐hospital cardiopulmonary resuscitation.

**Design:**

A qualitative descriptive design. Methods: Eighteen intensive care nurses from two regions and three hospitals in Sweden were interviewed. The data were analysed with General Inductive Analysis.

**Results:**

The work for intensive care nurses in the emergency team was reflected in three phases: prevention, intervention and mitigation—referred as before, during and after the CPR situation.

**Conclusions:**

The findings describe the complexity of being an intensive care nurse in an interdisciplinary emergency team, which entails managing advanced care with limited and unknown resources in a non‐familiar environment. The present findings have important clinical implications concerning the value of having debriefing sessions to reflect on and to talk about obstacles to and prerequisites for performing successful resuscitation.

## 1 INTRODUCTION

The immediate need for reallocation of staff and material resources to life‐threatening critical situations, such as cardiac arrest, has been recognized in the medical and nursing literature since the early 1990s under the term *code teams* (Blackhall, 1987; Molan, 2013; Whitcomb, 1990). Similar concepts in this field of research include “code blue,” “emergency code,” “hospital rapid response team” and “emergency teams” (ETs), which is the term used here. ETs are a service that can be called in emergency circumstances anywhere in the hospital. Further, ETs provide support in hospitals and are manned by regular staff, such as nurses and physicians, who are specially trained in anaesthesia and/or intensive care. Despite the well‐established concept of ETs, there are no strict policies about their design. However, the ETs hold a vital position in hospitals, as it is well‐known that successful cardiopulmonary resuscitation (CPR) outcomes correlate with two main components: the time elapsed between cardiac arrest and initiation of CPR and the quality of performance (Aune et al., 2011; Fredriksson et al., 2006; Herlitz et al., 2001). Competence among hospital staff in recognizing warning signs and the possibility to preventively treat patients at risk of cardiac arrest are critical in stabilizing cardiac care and increasing survival among those at risk (Deakin et al., 2010). A prerequisite for functioning ETs is that the different professional groups can communicate across the boundaries of their professional roles and form a team identity (Reeves et al., 2010).

## 2 BACKGROUND

Nurses are expected to manage heart failure using evidence‐based knowledge (Aune et al., 2011; Dwyer & Mosel Williams, 2002). Nonetheless, nurses describe being anxious about conducting CPR (Mäkinen et al., 2009). It has been reported that healthcare professionals appreciate CPR educational programmes using dummies and standardized protocols, as these give a sense of increased knowledge and confidence (Kozamani et al., 2012; Plodr et al., 2016). However, Mortell (2009) argues that scenario practice on dummies does not cover all aspects of CPR situations, especially the emotional experience, and refers to this problem as a theoretical–practical–ethical gap. Another factor that impacts staff anxiety and stress is preparation time. For example, prehospital care professionals have the advantage of being able to prepare themselves in advance, as they are briefed during transportation in the ambulance (Larsson & Engström, 2013). Many studies of CPR focus mainly on the biophysical/medical and technical aspects of the procedure and its development (Chen et al., 2014; Cho et al., 2014; Hasegawa et al., 2014). Some aspects were identified as important for reducing the outcomes of hospital mortality and cardiorespiratory arrest, for example, the team leader's critical skills, educational interventions and communication skills (cf. Maharaj et al., 2015; Winters et al., 2013). In a review article, McNeill and Bryden (2013) assessed the existing literature on whether early warning systems or multidisciplinary emergency response teams improve patient survival rates in hospitals. They found a high level of evidence that emergency teams reduce mortality rates and that teams reduce needs for transferring patients to ICUs. Interestingly, they recommend the adoption of a whole system approach, since a single‐parameter approach is limited. Further, McNeill and Bryden (2013) exhort that future research ought to focus on “the effect of a comprehensive emergency response process” (p. 1663). In response to that, Nilsson (2007) argued that the focus of clinicians should be on doing things correctly, rather than trying to achieve constant improvements by doing different and new things. In a previous article from the same research project, we published the findings of a qualitative investigation on nurses’ experiences of conducting CPR in ICUs (Sjöberg et al., 2015). We found that CPR situations were perceived as stressful, even for specialized nurses working in a high‐technological environment. During a CPR situation, there is an imminent need to bring order to the situation and avoid chaos. Thus, the need for a leader and the ability to communicate effectively and clearly are vital; if too many people are involved, it was considered to cause obstruction and lead to messiness. Although technical knowledge is critical for decreasing mortality rates, there is an urgent need to further study nurses’ subjective experiences of conducting CPR as part of an ET. Little research is published on the subjective experience of being part of a rapid response team. Few studies have used qualitative designs and looked at the problem from a process‐focussed perspective. The explicit aim of the present study was to describe intensive care nurses' reflections on being part of interdisciplinary emergency teams involved in in‐hospital cardiopulmonary resuscitation. The research question is: what reflections do intensive care nurses make from being involved in in‐hospital cardiopulmonary resuscitation in an interdisciplinary team?

## 3 METHODS

### 3.1 Research design

A qualitative study design was chosen for this project as the objectives were explorative and descriptive (Patton, 2015). In accordance with the chosen design, nurses were interviewed about their experiences. The overall research question that guided the study was to explore and describe how intensive care nurses' reflected on being part of interdisciplinary emergency teams involved in in‐hospital cardiopulmonary resuscitation. Data were analysed using the General Inductive Analysis, as described by Thomas (2006).

### 3.2 Settings and participants

Three hospitals in two regions of Sweden were selected using purposive and convenience sampling (Patton, 2015). Because the three hospitals varied in size, the response time also differed, but how the summoning to the alarming ward was organized was the same. One of the ICU nurses was responsible for holding the caller. When the alarm sounded, the team members met up and together moved to the alarming ward. The emergency team members had no specific education except their specialist training as professionals. However, prior to being part of the team, they had attended a few alarms as observers. Note that the constellation of the team members differs every day, and the team is composed of the professionals working on the particular shift. The inclusion criteria were as follows: (a) specialist nurses in intensive care, (b) working in the ICU at least 12 months, and (c) experiences of the alarm process, in their role as ET members during cardiac situations during the past 12 months. No specific exclusion criterion was set. None of the participants dropped out. No further specific participant characteristics were collected, as we were interested in their reflections on an aggregated level; we did, however, try to achieve variation in the participant group (Patton, 2015). The total sample was based on 18 interviews. Eight nurses participated from Hospital A, eight from Hospital B and two from Hospital C; there were two male and 16 female nurses. The nurses ranged in age from 29 to 64 years, and their average number of years of experience as specialist nurses in critical care was 15 years, ranging from 18 months to 25 years. Hospital A is a 500‐bed university hospital, Hospital B a 200‐bed regional hospital and Hospital C a 400‐bed university hospital.

### 3.3 Data collection

Qualitative semi‐structured interviewing is a highly suitable method for collecting data, as it yields deep and rich insights into the subjective phenomena of interest (Patton, 2015). We developed an interview guide based on the study aim, with inspiration from Spradley's (1980) ethnographic interview technique. Background questions were first asked concerning their experiences of being intensive care nurses and of in‐hospital CPR situations. The interviews then continued with open‐ended questions about descriptions of the team and descriptions of in‐hospital alarm situations. In designing the guide, we strived to pose both broader and more focussed questions. An example of a broad question is: “Can you please tell me the overall situations of what happens when the alarm sets of?” and an example of a more focussed question is: “Can you please tell me about a typical alarm situation? Also, in line with Spradley, we also posed contrast questions such as “What would you say is the difference between a CPR situation in the ICU and an in‐hospital situation?” During the interviews, which were held in a private room in the hospital during or after the work shift, an interview guide was used to ensure that all participants were asked the same questions; probing questions were more flexible. The interviews ranged from 17 to 61 min in duration (average 39 min). The second author, a highly experienced ICU nurse working in clinical practice, conducted all of the interviews.

### 3.4 Data analysis

We adopted an inductive analysis approach and made use of a specific, structured method called General Inductive Analysis (Thomas, 2006). In the first step, the audio files were transcribed. In the second step, the data were read and re‐read; meaning units (short sentences or paragraphs and containing claims) were extracted related to the study aim and stored in a separate Excel sheet. The meaning units were then condensed into shorter phrases; for example, the meaning unit “Most of the time you (*the person manning the heart pager*) are assigned a patient who can be left alone for perhaps a few minutes without someone having to monitor them constantly…” was condensed to “Assigned responsibility for a patient on one's own ward who can be transferred to someone else in case of an alarm.” In the third step, the meaning units were assigned codes; for example, the condensed meaning unit was given the code “To plan and prepare.” The codes were analysed and grouped into categories based on their similarities and differences; for example, we critically asked ourselves questions such as: How do the codes “To prepare oneself” and “Important traits for a nurse during ongoing emergency situations” differ? In the fourth step, codes in each category were examined and collated. Codes that did not fit into any of the categories were removed. Throughout this critical examination, we moved between the parts (codes) and the whole (categories), thus viewing the analysis from different perspectives. In the fifth and final step, the categories were analysed for their content and meaning and then compared with the study aim. To reach consensus on the categories, we engaged in in‐depth discussions. An example from the analysis is shown in Table 1. The three constructed categories are called phases in the results section and presented in chronological order.

**TABLE 1:** Example from the data analysis

Meaning unit	Condensed meaning unit	Code	Category
There are times when the heart pager goes off (*the alarm sounds*) and you really feel that no, I can't leave the room, because it's…if I have to change the noradrenaline all the time, and that's, I want to keep that responsibility, I don't want to give up the responsibility for this patient to someone who's “loose” in the corridor…	Occasions when the heart pager sounds the alarm and the nurse does not want to give up patient responsibility to another member of staff	Responsibility for the patient makes the nurse unwilling to transfer responsibility	Prevention phase
Most of the time you (*the keeper of the heart pager*) are assigned a patient who can maybe be left alone for a few minutes without someone having to monitor them constantly…	Assigned a patient responsibility on one's own ward that can be transferred to someone else in case of an alarm	To plan and prepare	Prevention phase
You try to get a picture of what is this, what do we have in front of us?	Create a picture of the situation before arriving on the scene	To prepare	Prevention phase
When you are running around on different wards you have to be quite humble and kind of see for those who have started it and pushed the button, they shouldn't have to feel like they are less in any way…we're just supposed to be a unit that arrives and gives support and doesn't take over in any way, a helping resource.	Important to be humble as a person, give support and help	Important traits for the nurse during ongoing emergency situations	Intervention phase
That (*the protocol*) is the entire lifeline, I think. Without the protocol you lose track of time really quickly. I think it's great that you can…you don't have to make a note of the time, you can just check it off that I have done this if you feel that it's too much to write down a time, so you know where you are in the program.	The protocol is the lifeline and helps you to not lose your place in the programme	The protocol is a prerequisite and a support in the emergency situation	Intervention phase
And perhaps I will think about the ethics, how will this 85‐year‐old lady recover after the cardiac arrest and after these compressions, and this possible lack of oxygen that she has been affected by…	How will this elderly woman be able to recover from what has happened during the cardiac arrest	Ethical reflections after cardiac arrest	Mitigation phase
When you have been working for a while and you have been running on a few alarms you can sort of see more outside yourself and say, it was good that you did that and you functioned really well…I think that might mean a lot to those who were there.	Experienced nurses give more feedback, which might mean a lot to those involved in the situation	Valuable for those involved to receive feedback after completed alarm	Mitigation phase
But otherwise I think that it's quite common that you don't talk, you go back and then you continue with your work tasks as if nothing has happened.	You don't talk about everything, you are supposed to keep working after CPR as if nothing has happened	Expected to go back to work as if nothing has happened	Mitigation phase

### 3.5 Ethical considerations

The project was approved by the Regional Ethical Review Board (Dnr.2016/015). Prior to the interview, all participants received both verbal and written information about the study; they were told that participation was voluntary and that they could withdraw at any time without further explanation. They then gave their written consent.

### 3.6 Rigour

Different techniques were used to ensure the trustworthiness of the study (Patton, 2015). All interviews were conducted by one of the authors using the interview guide. The data analysis was performed by the author AO. Thus, the final steps of the analysis and the results were then discussed by all members of the research group. The data analysis process is clearly described in the methodology section of the present study to ensure credibility. Several illustrative quotes were also linked to the categories in the results to enhance the study's credibility.

## 4 RESULTS

Nurses in the ETs reflected on being part of rescue situations in an interdisciplinary emergency team. Based on our analysis, three phases emerged: prevention (before the CPR situation), intervention (during the CPR situation) and mitigation (after the CPR situation).

### 4.1 Nurses’ reflections on being part of the rescue situation in the prevention phase

The nurses reflected on how they prepared the upcoming shift. Preparation meant, that is, being able to, on short notice, make one nurse on the ward the “loose one” (ICU nurse 9), hence the ICU nurse for the ETs. This nurse carried the pager during the working shift, answered the alarm and was part of the ET. The nurses reported that being the loose one meant being prepared to just “take off” (ICU nurse 5), leaving one's patients, duties and responsibilities to colleagues: “when you're forced to respond to the alarm it sometimes feels like you're leaving your patients to fend for themselves” (ICU nurse 12). Leaving patients immediately was sometimes nearly impossible. In such circumstances, the nurse needed to either find a colleague, experienced enough to either take over the pager or take care of the patient. Nurses described “leaving” as stressful, thus not being able to free oneself immediately or quickly find someone else to join the ET. One important aspect of preparation described by several nurses was having good knowledge of the hospital locality: “maybe you don't need to be able to find your way flawlessly in the hospital, you can receive instructions, but it's important that you are able to orient yourself” (ICU nurse 10). In the interviews, the nurses reported that the ET and its equipment were to be at the ward sounding the alarm in approximately three minutes—a goal the ETs met most of the time. The hospitals were constructed differently, and the limited amount of time to reach the ward sounding the alarm meant that nurses needed to be up to date on the emergency routes. Building renovation was described as an obstacle: “so there we come rushing in with all kinds of stuff…and suddenly it's just like a stop…like, a construction site…” (ICU nurse 8). The ETs used maps of the hospital buildings to help them.

Preparation was not done just for daily work, but could also involve having to educate new and less experienced colleagues at the ICU as well as healthcare staff on other wards in being prepared for an emergency situation. Another kind of prevention reflected on by the nurses was the responsibility for establishing “step‐by‐step guides” for other wards clarifying what to do and how they could help the ET during the “rescue action.”

### 4.2 Nurses’ reflections on being part of the rescue situation in the intervention phase

The nurses reported that one obstacle in the physical work environment was the equipment they had to bring with them to the site of the alarm. It was heavy and cumbersome, creating yet another obstacle that made it difficult to move quickly to the ward sounding the alarm. The emergency backpacks were described as big and unwieldy, “that backpack is damn clumsy…it would…you could minimize that in many ways…I don't think it's entirely easy to run with” (ICU nurse 2), and the scooters the ETs used were difficult to manoeuvre. The nurses also described obstacles related to the pager, which has a very small display. This made it very difficult to see what had happened (on the ward sounding the alarm). The information on the screen only showed where they were supposed to go. Once on site, they received information on what had happened and what the ward sounding the alarm needed help with. Upon arrival, another obstacle was crowding, that is, all of the staff, relatives and other things in the patient's room. In this connection, it might be necessary to leave some equipment in the corridor, especially the emergency scooter. The greatest difficulty was said to be on the psychiatric ward, which almost completely lacked standardized emergency care equipment. Staff and/or relatives not directly involved in the CPR work could sometimes become passive observers. The nurses might have felt that their presence reduced the procedure's efficacy, “And then I think that relatives should be…gently removed from the room and someone has to take care of them…in order to create space…above all, remove the stress factor [for the staff]” (ICU nurse 11).

An important qualification for a nurse in an ET was being flexible in the emergency situations, being able to back‐up/support colleagues in some parts of the CPR procedure. An obstacle to achieving successful resuscitation described by the nurses was the lack of clear leadership, delegation of work tasks and communication with all involved parties. Formally, this is the responsibility of the medical doctor or anaesthesiologist. In circumstances when the physician was unexperienced or could not manage the situation, several nurses reported having been forced to assume responsibility during the resuscitation. In all CPR actions, a protocol is used to maintain a structure during the procedure: “you can really feel unsure of yourself, am I thinking about this wrong, then you look at the paper [the protocol]…” (ICU nurse 14). Administering drugs and signing the protocol were described as the most important things for the nurses to do during CPR. The nurses also reported feeling that they needed to notice other staff members’ stress and try to reduce it by, for example, ensuring staff rotation, encouraging communication and ensuring that physicians made decisions based on the protocol. Arriving on wards where CPR was used more frequently, thus where staff were used to CPR situations and familiar with the CPR protocol, the interviewed nurses reported feeling superfluous. Some alarms were more associated with emotions, and paediatric wards were particularly stressful: “when I saw that there were children, then I felt anxious, because even if you've worked a while children are the most difficult to deal with, it really gets to you.” (ICU nurse 6), “when you're running to a child [alarm] then…then you have time to think that we will damn well not mess this up” (ICU nurse 10) and “so then I thought, I won't look at the face of the girl…I’ll just concentrate on what I’m doing…but then as it happens…it's impossible” (ICU nurse 3).

### 4.3 Nurses’ reflections on being part of the rescue situation in the mitigation phase

The final phase involves describing what happens when the rescue action is over, whether or not the patient survived. The nurses reported a lack of debriefing after the rescue; however, none of the nurses said that they really needed debriefing. However, sometimes they felt they adjusted to other staff members’ (from wards sounding the alarm) need for debriefing: “we sometimes help out when others need to talk, but we manage on our own.” (ICU nurse 17) However, when the nurses had been called out to the paediatric ward and a child had died, they expressed a greater need to debrief: “when that child died it would have been good to talk to someone” (ICU nurse 9). All nurses expressed a need to talk about a CPR situation that had been emotional, for example, if the patient had died, especially a child. Hence, no formal debriefing has been performed, as one nurse said: “generally you just have to clench your teeth and keep going, just shake it off, that's how it is” (ICU nurse 18). Instead, brief informal conversations with colleagues took place when there was an opportunity, in the break or medications room. These conversations took place entirely on individuals’ own initiative. Conversations with the physician participating in the CPR could take place briefly in, for example, the elevator on the way back, because there was rarely time to talk about it again when they returned to the ICU. One nurse said: “those of us who work as ICU‐nurses are supposed to be hardened,” (ICU nurse 3) and another nurse reported: “You do what you're supposed to do and then you move on to the next thing, no time to think…” (ICU Nurse 13). One obstacle to finding time to talk was the pressure the nurses felt to return to their duties and responsibilities at the ICU: “you need to catch up with what you left…” (ICU nurse 11). Some nurses had experiences of organized debriefing conversations on other wards and found them very helpful, but one prerequisite was that debriefing take place close in time to the CPR event.

The nurses reported having discussed some circumstances they had experienced during the year as well as ethical considerations that had arisen during the annual CPR training. Depending on the patient's age and disease history, performing CPR sometimes felt like a degrading extension of his/her death.

One of the obstacles described was that the ET members do not know the patient and the physician responsible in the CPR situation does not have time to read the patient's journal. In some situations, the nurses felt that an undignified death could have been prevented if a treatment plan had existed. The view, however, was that such a plan seldom exits, as the duty to save life might conflict with feelings of human compassion, where duty is superordinate to feelings: “sometimes it feels like we are treating a corpse…” (ICU nurse 1). Some nurses also reported having felt like “a horrible person” (ICU nurse 15) when they did not let an old, severely ill person die with dignity, and that this is particularly problematic with unexperienced physicians, who do not dare make the decision to interrupt treatment even when all hope is gone, “we have different perspectives, nurses and physicians, on the ethical part. We think a bit differently…you don't enter into any ethical discussions…you leave that for the physicians…me, I just do my job” (ICU nurse 7).

## 5 DISCUSSION

The present study was designed to describe intensive care nurses' reflections on being part of interdisciplinary emergency teams involved in in‐hospital cardiopulmonary resuscitation. The results were structured in three chronologically ordered phases: prevention, intervention and mitigation—which occurred before, during and after the CPR situation. During the first phase—prevention—the most prominent feature was the stress of leaving one's colleagues and ICU patients, even though there was an agreement between team members and the remaining ICU nurses. We believe this might be connected to the sense of ethics and morality that is inherent in the nursing profession (Kangasniemi et al., 2015; Liaschenko & Peter, 2004; Milliken, 2018). The nurses reflected on the fact that being called out to an in‐hospital CPR situation was played down as being nothing more than “part of the job” and associated with an insignificant emotional impact on nurses’ working life. The results concerning the first phase demonstrate that reflections were more related to stress deriving from interdisciplinary teamwork than to the resuscitation itself (Barr, 2014). As indicated by Schot et al. (2020), it is vital to overcome interprofessional gaps and create a space for the special skillset of each profession. Overall, this study strengthens the idea of the importance of emergency teams to meet and discuss teamwork and team members’ respective roles. Thus, creating a united team characterized by respect for each other's competence and roles across professions may lead to increased patient safety (cf. Hamilton et al., 2017)—although, this requires further investigation.

Previous research has stressed the importance of teamwork, but also emphasized some of its inherent challenges (Barr, 2014; Grover et al., 2017; Nancarrow et al., 2013). Teamwork was considered especially important during the second phase: the intervention phase. Fundamental to a team in which several specialties collaborate under pressure, and with members who have not previously worked together, is clear leadership (Hunziker et al., 2011; O’Donoghue et al., 2015), something also reflected on by the nurses as especially important and a prerequisite for a functional work environment. Physicians’ inability to lead, delegate tasks and communicate in a stressful situation such as CPR may result in chaotic situations and unwanted outcomes (Sjöberg et al., 2015). Furthermore, the nurses’ reflections on failed leadership were primarily related to situations lacking clear frameworks and limits on the respective professions’ responsibilities (cf. Wood et al., 2020). One way to strengthen confidence and increase nurses' skills is to use simulation training (Andreatta et al., 2011). Having confidence and leadership skills has also been shown to increase patient safety (Ballangrud et al., 2014). The results also indicated a lack of organized debriefing conversations in connection with the CPR situation. Two studies have pointed out the need for debriefing after events such as CPR (Clark & McLean, 2018; O’Donoghue et al., 2015) as a way of processing experiences and reflecting on the situation. The organized talks that several researchers have advocated (Clark & McLean, 2018; O’Donoghue et al., 2015) may, however, not be sufficient if the situation was very dramatic. For example, situations involving children dying are particularly difficult to “let go of”—as seen in the present results. The failure to allocate time to debriefing sessions was mentioned as an obstacle. According to Huggard (2013), a conversation lasting only a few minutes may be sufficient and could take place before the work shift is over. Corbett et al. (2012) suggest that a systematic way to evaluate one's efforts in a situation is to listen to others, where one is allowed to process what one has experienced. Organized conversations can provide more than mental relief and may also be a way to improve one's skills. The group may also be able to evaluate their efforts in order to improve individual and team performance (Couper & Perkins, 2013).

Every CPR situation gives rise to ethical considerations in one way or another (Mentzelopoulos et al., 2018). Our interpretation of the present results is that the nurses incorporated a biomedical outlook, setting priorities as to which form of symptom relief was most crucial in conjunction with CPR. Furthermore, they reflected on the ethical issues that could arise when performing CPR on a patient and possibly result in unnecessary suffering for the patient (Lee & Cha, 2018). De Gendt et al. (2007) highlight the importance of nurses being seen and being part of the discussion when decisions are made on possible treatment restrictions. Aune et al. (2011) means that nurses have an ethical responsibility and should, therefore, be a natural part of such discussions.

### 5.1 Study limitations

One potential weakness is that the interviews were conducted retrospectively; thus, the participants may have forgotten important information. However, we reason that the participants have chosen to narrate memorable situations, which can be considered meaningful information for them and, thus, in alignment with the study aim. Yet all interviews were performed by the second author (F.S.), an ICU nurse himself. Conducting research in a familiar setting may cause bias and pre‐assumptions about the phenomena under study as well as participant expectations of the researcher/interviewer (Asselin, 2003). Researcher reflexivity is also a matter of importance to address when conducting research in a familiar field (Etherington, 2004). Between interviews, F.S. discussed the quality of the interviews with the last author, M.S‐E. Even though F.S. posed naïve probing questions, some information might have been overlooked due to F.S.’s insider perspective. However, each interview was analysed by the first author (AO), and the analyses were discussed among the whole research group, ensuring the rigour of the research process. Due to the qualitative aspect of transferability, we do not claim that the present results are applicable in other settings. On the other hand, because we included participants from different wards to increase the variation in the sample, we can on good grounds assume that the reported experiences are relevant to other rescue team members (Creswell & Poth, 2018).

## 6 CONCLUSION

In conclusion, the findings of the present interview study reveal three phases: before, during and after the CPR situation. Our study provides insights into the complexity of being an ICU nurse in an ET, given the obstacles and prerequisites that may exist in the work environment. We conclude that preparation and adherence to the protocol are vital. Additional conclusions are that CPR situations may have an emotional impact on nurses, and that functional teamwork is a necessity. Hence, there is a need for acknowledgement and debriefing sessions. The findings may provide a basis for improved team development, based on a mutual understanding of each profession´s role, but also improved interdisciplinary communication. As to the present study's clinical implications, we believe clinical development nurses and managers can make use of the results and introduce debriefing sessions. In addition, we suggest that members of ETs meet on a regular basis to discuss obstacles and prerequisites for their practices. Future research might also focus on how information and communication technologies (ICT) and artificial intelligence (AI) could support the ICU nurses’ working environment.

## DECLARATIONS

Ethics approval and consent to participate. The project was approved by the Regional Ethical Review Board (2016/015).

## CONSENT FOR PUBLICATION

All participants gave their written consent for publication.

## CONFLICT OF INTEREST

The authors declare that they have no competing interests.

## AUTHORS CONTRIBUTIONS

FS and MSE were responsible for the design of the study. AO analysed the data and wrote the Results and parts of the Discussion. FS collected and transcribed the data, participated actively in the final steps of the data analysis and wrote parts of the Background. MSE analysed the data and wrote parts of the Background, Methods and parts of the Discussion.

## Data Availability

The data sets generated and analysed during the current study are not publicly available due to the need to protect the participants’ personal integrity. Thus, the data sets are safely stored at the University of Gävle.
